# Incidence and Risk Factors of Hearing Impairment in Full-Term Neonatal Intensive Care Unit (NICU) Graduates: A Cross-Sectional Observational Study From Central India

**DOI:** 10.7759/cureus.90399

**Published:** 2025-08-18

**Authors:** Yogesh Pehlajani, Jyotsna Shrivastava, Shipra Mandraha, Amit Agrawal, Monali Datta

**Affiliations:** 1 Pediatrics, Netaji Subhash Chandra Bose Medical College, Jabalpur, IND; 2 Pediatrics, Gandhi Medical College, Bhopal, IND

**Keywords:** bera, dpoae, full-term neonates, hearing loss, nicu graduates, risk factors

## Abstract

Background: Early hearing screening of full‑term neonates discharged from the neonatal intensive care unit (NICU) is essential to enable timely interventions that support optimal language and cognitive development. Despite their potential vulnerability, this population has received limited attention compared with preterm infants, underscoring the need to establish the incidence of hearing impairment and identify key maternal and neonatal risk factors. The primary objective of this study was to determine the incidence of hearing impairment among full-term neonates discharged from the NICU. The secondary objective was to assess the association of various maternal and neonatal risk factors with the occurrence of hearing loss.

Methods: This prospective, observational, cross-sectional study was conducted at the Department of Pediatrics, Gandhi Medical College, Bhopal, India, from September 2022 to March 2024. A total of 229 full-term NICU graduates were enrolled using predefined inclusion criteria. Hearing screening was conducted using distortion product otoacoustic emissions (DPOAE) as the initial and repeat test, followed by brainstem evoked response audiometry (BERA) for neonates who failed the second DPOAE. Data were analyzed using IBM SPSS Statistics software, version 25 (IBM Corp., Armonk, NY), employing descriptive statistics and the chi-square/Fisher's exact test for associations.

Results: Of the 229 neonates screened, three were found to have confirmed hearing impairment based on BERA, yielding an incidence of 13 per 1,000 NICU graduates. Significant associations were noted between hearing impairment and risk factors such as meningitis (p=0.0262), hypoxic-ischemic encephalopathy (HIE) (p=0.0044), bilirubin encephalopathy (p=0.022), oligohydroaminos (p=0.0001), and ototoxic medication use (>5 days) (p=0.049).

Conclusion: In conclusion, the study highlights that full-term NICU graduates are at risk for hearing impairment and require the need for routine and comprehensive auditory screening. Early detection enables timely intervention, thereby reducing long-term disability.

## Introduction

Hearing loss is one of the most prevalent congenital sensory impairments globally, with profound implications for cognitive, linguistic, and psychosocial development. According to the World Health Organization (WHO), approximately 63 million individuals in India suffer from disabling auditory impairment, underscoring the substantial burden of this condition in a single low- and middle-income country. Globally, the estimated incidence of permanent bilateral hearing loss is one to three per 1,000 live births in otherwise healthy term neonates. However, this risk increases significantly among high-risk populations, particularly those admitted to neonatal intensive care units (NICUs), where studies report incidences as high as 20 to 40 per 1,000 live births [1]. The increased susceptibility in these infants is often linked to conditions such as hypoxic-ischemic encephalopathy (HIE), hyperbilirubinemia, neonatal sepsis, craniofacial anomalies, and exposure to ototoxic medications, all of which can result in irreversible cochlear or auditory nerve damage. In the Indian context, a review by Verma et al. (2021) revealed that the incidence of neonatal hearing impairment ranges from 1.59 to 8.8 per 1,000 births in the general population, with substantially higher rates ranging from seven to 49 per 1,000 births among neonates with identifiable risk factors. Despite such compelling evidence, universal newborn hearing screening (UNHS) remains inconsistently implemented in India, particularly in under-resourced settings [2].

While extensive research has focused on hearing loss among preterm neonates and those with major comorbidities, full-term infants admitted to NICUs constitute an important yet underrecognized high-risk group. Term neonates experience insults similar to preterm neonates, like sepsis, meningitis, hyperbilirubinemia, and ototoxic drug exposure, but remain underrepresented in neonatal auditory health research. This gap is particularly evident in low- and middle-income countries, where systematic surveillance is often lacking [3]. 

The advent of objective screening tools such as otoacoustic emissions (OAE) and brainstem evoked response audiometry (BERA) has significantly improved early detection of neonatal hearing loss. OAE serves as a rapid, noninvasive, and cost-effective screening tool, while BERA offers a more definitive diagnostic evaluation with high sensitivity and specificity. A two-stage protocol involving both modalities enables timely identification and referral, thereby enhancing neurodevelopmental outcomes. Recognizing the high prevalence of modifiable risk factors and the irreversible consequences of delayed diagnosis, this study was conducted to estimate the incidence of hearing impairment among full-term neonates discharged from NICU and to examine the association of maternal and neonatal morbidity factors with the development of hearing loss in this vulnerable population.

## Materials and methods

This study was designed as a prospective, observational, cross-sectional study conducted in the Department of Pediatrics at Gandhi Medical College and Associated Hospitals, Bhopal, India. The study was conducted over an 18-month period from September 2022 to March 2024. The target population included full-term neonates (≥37 weeks’ gestation) who were admitted to the NICU and were discharged alive in stable condition. The sample size was calculated using the following standard formula for estimating a proportion in a cross-sectional study:

$n = \frac{Z^2 p (1 - p)}{d^2}$

where Z is the Z-score corresponding to the 95% CI (1.96), p is the estimated prevalence of hearing impairment (18%) taken from a study conducted by Rawat et al. [4], and d is the desired absolute precision (5%). Substituting the values, a final sample of 229 neonates was included using a total enumeration technique, where all eligible neonates admitted during the study period were enrolled consecutively. Eligibility was determined by predefined inclusion and exclusion criteria. Neonates were included if they were full-term, admitted to the NICU, and discharged after recovery. Neonates were excluded if they had a family history of congenital deafness or external ear anomalies, or if informed consent was not provided by parents or guardians.

Data were collected using a pre-tested, semi-structured questionnaire developed by the investigators. The tool captured maternal obstetric history (e.g., gravida status, pregnancy-induced hypertension) and neonatal clinical variables (e.g., birth weight, sepsis, hyperbilirubinemia, perinatal asphyxia, meningitis, ototoxic drug exposure). Pre-testing of the tool was conducted on 10 neonates outside the study sample to refine language, sequence, and cultural appropriateness. Modifications were made accordingly to improve clarity and reliability. Data collection was performed by a trained pediatric resident under the supervision of faculty. Training sessions were held to standardize procedures related to history-taking, questionnaire administration, and auditory testing.

Each neonate underwent hearing screening in a soundproof room using distortion product otoacoustic emissions (DPOAE) as a first-level test prior to discharge. A "pass" was defined as ≥3 frequencies passed out of four (2, 3, 4, and 5 kHz) in each ear. A "refer" in either ear was considered a failed screen. Infants who passed or failed the first screening both underwent a repeat DPOAE after two weeks. Persistent failures were subsequently evaluated using BERA, with responses interpreted by a trained audiologist. The BERA used click stimuli at 35-45 dB with standard electrode placement, and abnormal results were defined as delayed or absent waveforms beyond age-appropriate latency thresholds. Calibration of the DPOAE and BERA instruments was conducted quarterly as per manufacturer guidelines. Internal quality control measures included routine pre-use checks, environmental noise assessment, and electrode impedance monitoring.

Data were initially recorded on printed proformas and later entered manually into Microsoft Excel 2016 (Microsoft Corp., Redmond, WA). To ensure data quality, 10% of records were randomly cross-verified by an independent supervisor. Incomplete or ambiguous entries were flagged and resolved by reviewing source documents. Ethical approval for the study was obtained from the Institutional Ethics Committee of Gandhi Medical College, Bhopal (Ethical Committee approval number 32107/MC/IEC/2022). Written informed consent was obtained from the parents or legal guardians of all participants. Confidentiality of all personal identifiers was strictly maintained, and anonymized data were used for analysis. Statistical analysis was performed using IBM SPSS version 25 (IBM Corp., Armonk, NY, USA). Categorical variables were expressed as frequencies and percentages. The association between risk factors and hearing impairment outcomes (based on DPOAE and BERA results) was assessed using the chi-square test or Fisher’s exact test, as appropriate. A p-value of <0.05 was considered statistically significant.

## Results

A total of 229 full-term neonates admitted to the NICU were enrolled and successfully underwent the complete sequence of auditory screening, including DPOAE and, where indicated, confirmatory BERA testing. The findings below summarize the screening outcomes, incidence of hearing loss, and the association of maternal and neonatal risk factors with auditory impairment. As shown in Table 1, the first-stage DPOAE screening revealed that 192 neonates (83.8%) had a bilateral pass result, while 37 neonates (16.2%) showed either a unilateral or bilateral refer outcome. The second-stage (final) DPOAE screening showed that seven neonates were again referred. None of the neonates showed unilateral referral at the second screening stage.

**Table 1:** Hearing screening outcomes at primary and final otoacoustic emissions (OAE) stages

Screening stage	Total Screened (n)	Pass %	Refer %
Primary (first) OAE	229	192 (83.8%)	37 (16.2%)
Final (second) OAE	229	222(97.0%)	7 (3 %)

The seven neonates who failed the second OAE screening underwent BERA for diagnostic confirmation. As summarized in Table 2, BERA identified hearing impairment in three out of the seven referred neonates, yielding a diagnostic yield of 42.8% among OAE-referred cases. The incidence of confirmed hearing loss in the total study population was therefore 13 per 1,000 neonates.

**Table 2:** Brainstem evoked response audiometry (BERA) diagnostic outcomes among final otoacoustic emissions (OAE) referred cases

BERA result	Frequency (n)	Percentage (%)
Abnormal	3	42.80%
Normal	4	57.20%
Total	7	100.00%

To identify potential determinants of initial screening failure, associations between maternal and neonatal risk factors and primary DPOAE outcomes were examined using chi-square and Fisher’s exact tests. Statistically significant associations were observed for the presence of oligohydramnios (p = 0.0001), HIE (p = 0.0044), meningitis (p = 0.0262), bilirubin encephalopathy (p = 0.0227), and prolonged use of ototoxic medications (p = 0.0493) (Table 3). In contrast, factors such as consanguinity, mode of delivery, pregnancy-induced hypertension (PIH), neonatal hyperbilirubinemia (NNH), and birth weight did not demonstrate statistically significant relationships with initial OAE outcomes.

**Table 3:** Association of risk factors with hearing impairment on primary otoacoustic emissions (OAE) screening (n = 229) *p-value was obtained using the chi-square test.

Variable	Category	Total	First sitting (pass)	First sitting (refer)	Chi-square	p-value*
Consanguinity	No	217	182	35	0.002	0.9608
	Yes	12	10	2		
Delivery type	Lower (uterine) segment cesarean section (LSCS)	74	62	12	<0.001	0.9867
	Normal vaginal delivery (NVD)	155	130	25		
Pregnancy-induced hypertension (PIH) history	No	194	165	29	1.363	0.243
	Yes	35	27	8		
Oligohydramnios	No	219	188	31	14.773	0.0001
	Yes	10	4	6		
Gender	Female	90	78	12	0.869	0.3512
	Male	139	114	25		
Weight group (kg)	<2 Kg	31	23	8	2.439	0.2954
	2-2.5 Kg	77	66	11		
	>2.5 Kg	121	103	18		
Perinatal asphyxia	Absent	193	159	34	1.922	0.1657
	Present	36	33	3		
Hypoxic-ischemic encephalopathy (HIE)	Absent	204	176	28	8.121	0.0044
	Present	25	16	9		
Sepsis	Absent	123	98	25	3.393	0.0655
	Present	106	94	12		
Meningitis	Absent	212	181	31	4.943	0.0262
	Present	17	11	6		
Neonatal hyperbilirubinemia (NNH)	Absent	202	170	32	0.118	0.7316
	Present	27	22	5		
Bilirubin encephalopathy	Absent	228	192	36	5.189	0.0227
	Present	1	0	1		
Ototoxic medication (> 5days)	Absent	161	140	21	3.863	0.0493
	Present	68	52	16		

Further analysis of the risk factors in the second-stage OAE screening showed that HIE (p = 0.0060), meningitis (p = 0.0003), and bilirubin encephalopathy (p < 0.0001) were significantly associated with persistent refer status (Table 4). Other variables, including sepsis, ototoxic drug use, gender, and birth weight, were not significantly associated with final screening failure.

**Table 4:** Association of risk factors with hearing impairment on final otoacoustic emissions (OAE) screening *p-value was obtained using the chi-square test.

Variable	Category	Total	Second sitting (pass)	Second sitting (refer)	Chi-square	p-value*
Consanguinity	No	217	210	7	0.398	0.5284
	Yes	12	12	0		
Delivery type	Lower (uterine) segment cesarean section (LSCS)	74	73	1	1.068	0.3013
	Normal vaginal delivery (NVD)	155	149	6		
Pregnancy-induced hypertension (PIH) history	No	194	188	6	0.006	0.9407
	Yes	35	34	1		
Oligohydramnios	No	219	213	6	1.694	0.1931
	Yes	10	9	1		
Sex	Female	90	88	2	0.347	0.5558
	Male	139	134	5		
Weight group (Kg)	<2 Kg	31	31	0	1.525	0.4666
	2-2.5 Kg	77	75	2		
	>2.5 Kg	121	116	5		
Perinatal asphyxia	Absent	193	186	7	1.341	0.2469
	Present	36	36	0		
Hypoxic-ischemic encephalopathy (HIE)	Absent	204	200	4	7.541	0.006
	Present	25	22	3		
Sepsis	Absent	123	121	2	1.828	0.1764
	Present	106	101	5		
Meningitis	Absent	212	208	4	13.134	0.0003
	Present	17	14	3		
Neonatal hyperbilirubinemia (NNH)	Absent	202	196	6	0.041	0.8393
	Present	27	26	1		
Bilirubin encephalopathy	Absent	228	222	6	31.714	<0.0001
	Present	1	0	1		
Ototoxic medication (>5 days)	Absent	161	157	4	0.597	0.4399
	Present	68	65	3		

## Discussion

In this prospective study of 229 full-term NICU graduates, the incidence of confirmed hearing impairment diagnosed by BERA was 13 per 1,000 neonates. The implementation of a two-stage screening protocol using DPOAE, followed by BERA for referred cases, proved effective in reducing false positives and identifying true cases early. Significant associations were observed between hearing impairment and specific neonatal risk factors, including HIE, meningitis, bilirubin encephalopathy, oligohydramnios, and prolonged exposure to ototoxic medications. These findings underscore the heightened auditory vulnerability in NICU-admitted term neonates and reinforce the value of integrating structured auditory screening protocols into standard neonatal care. A two-stage screening protocol was employed to account for transient factors that can affect the accuracy of initial DPOAE results. Conditions such as environmental noise, neonatal movement, cerumen or vernix in the ear canal, and middle ear fluid can cause false positives. Additionally, respiratory interventions like continuous positive airway pressure (CPAP) or intubation may induce temporary conductive hearing loss through Eustachian tube dysfunction or barotrauma. Ototoxic medications may also cause reversible cochlear changes before leading to permanent damage. By repeating the screening after two weeks, many of these transient influences are likely to resolve, thereby improving test specificity and minimizing unnecessary diagnostic referrals. This approach ensures greater diagnostic precision, especially in resource-constrained settings.

The present study assessed the incidence of hearing impairment and its association with maternal and neonatal risk factors among full-term neonates discharged from a NICU. The confirmed incidence of sensorineural hearing loss (SNHL) in this cohort was 13 per 1,000, which is higher than the incidence in the general newborn population but aligns with figures reported among NICU-admitted neonates. For example, Pourarian et al. [5] observed a 13.7% prevalence of hearing loss in NICU neonates using otoacoustic emissions and auditory brainstem response (ABR) testing. Similarly, Aradhana et al. (2020) [6] reported that 13.7% of premature infants in their NICU cohort showed hearing impairment. While our cohort consisted exclusively of full-term neonates, the incidence remains within this high-risk range, suggesting that NICU admission itself, regardless of gestational age, marks increased vulnerability, likely due to cumulative exposure to intensive interventions and pathological insults [7].

One of the most significant findings in our study was the strong association between HIE and hearing screening failure. This finding is consistent with that of Dhande et al. (2023) [8], who identified neonatal asphyxia and HIE as significant predictors of SNHL in NICU graduates. The biological plausibility lies in the sensitivity of the cochlea and auditory brainstem to hypoxic injury; the stria vascularis and cochlear hair cells are particularly vulnerable to oxygen deprivation, leading to both metabolic and structural damage. Additionally, hypoxia may compromise central auditory pathways, thereby impairing neural transmission even in the presence of intact cochlear function [9].

Another notable risk factor in our cohort was meningitis, which showed a significant association with both initial and final OAE screening failures. This observation is in line with the findings of Khairy et al. (2018) [10] and Choi et al. (2020) [11], both of whom reported a strong link between neonatal meningitis and subsequent hearing impairment. The pathophysiology involves bacterial invasion of the cochlear aqueduct and internal auditory canal, leading to direct neuronal damage, fibrosis, and ossification of the cochlea, a well-recognized sequela of bacterial meningitis in neonates [12].

Bilirubin encephalopathy also emerged as a significant determinant of hearing screening failure in our study. This is consistent with previous literature, including Nair et al. (2018) [13], who found that hearing loss was more frequent among neonates with multiple risk factors, particularly those with hyperbilirubinemia and neurologic manifestations. The neurotoxic effects of unconjugated bilirubin on the auditory brainstem nuclei, particularly in the inferior colliculus and cochlear nucleus, are well established. The selectivity of this damage underscores the importance of prompt management of jaundice and routine auditory monitoring in affected neonates [14].

Also, while prolonged use of ototoxic medications (>5 days) was significantly associated with initial screening failure, this association did not persist in the final screening stage. This discrepancy may reflect transient auditory dysfunction that resolves upon cessation of the offending agent or may indicate subclinical injury insufficient to cause persistent sensorineural loss. This contrasts with findings from Sun et al. (2003) [15], who noted a 41.3% hearing loss rate among neonates exposed to ototoxic agents. The difference may be attributed to variations in dosing protocols, drug combinations, and concurrent use of protective strategies such as serum drug level monitoring. Finally, oligohydramnios was identified as a statistically significant risk factor in our study, but this has not been widely reported in other studies. The potential mechanism could involve intrauterine hypoxia and placental insufficiency affecting fetal auditory development [16]. However, the lack of replication in external datasets may suggest a population-specific finding or a spurious association influenced by confounding variables. Future research with larger sample sizes and stratified risk models could clarify the significance of this factor.

The present study’s demonstration of a 13 per 1000 incidence of SNHL in full-term NICU graduates underscores that this cohort can no longer be considered “low risk” and should therefore be incorporated into universal newborn hearing-screening (UNHS) programmes that already target preterm and syndromic infants. A two-stage protocol of bedside DPOAE followed by confirmatory BERA offers high sensitivity with a manageable false-positive rate and is consistent with international best practice for high-risk neonates [1]. The strong links shown here between SNHL and HIE, neonatal meningitis, and bilirubin encephalopathy mirror large population-based data demonstrating a several-fold increase in permanent hearing impairment after asphyxia-related encephalopathy, bacterial meningitis, and severe hyperbilirubinemia. Because irreversible cochlear and brainstem injury can occur within days, early diagnosis and entry into habilitation before six months of age remain critical [17]; children who meet Early Hearing Detection and Intervention (EHDI) timelines consistently achieve superior language, cognitive, and social outcomes [18]. In resource-constrained settings such as India, integrating structured hearing assessment into NICU-discharge protocols together with targeted follow-up of infants exposed to ototoxic drugs or perinatal hypoxia represents a feasible, evidence-based strategy to mitigate the lifelong burden of preventable deafness.

## Conclusions

This study highlights a notable incidence of hearing impairment among full-term neonates admitted to the NICU, emphasizing the importance of routine and comprehensive auditory screening even in non-preterm populations. The findings demonstrate that specific neonatal and perinatal risk factors like HIE, meningitis, and bilirubin encephalopathy are significantly associated with early hearing screening failure. These results underscore the need for vigilant monitoring and early intervention in neonates with such risk profiles. Incorporating a structured two-stage OAE screening protocol followed by confirmatory BERA testing prior to discharge can facilitate timely diagnosis and improve long-term neurodevelopmental outcomes. Future research involving multicenter cohorts and extended follow-up is warranted to validate these findings and capture delayed-onset auditory deficits.
